# Supplementary figures and images for: PASTA kinase-dependent control of peptidoglycan synthesis via ReoM is required for cell wall stress responses, cytosolic survival, and virulence in Listeria monocytogenes
Source: PLoS Pathog. 2021 Oct 8;17(10):e1009881. doi: 10.1371/journal.ppat.1009881 (PMC8528326; doi:10.1371/journal.ppat.1009881)

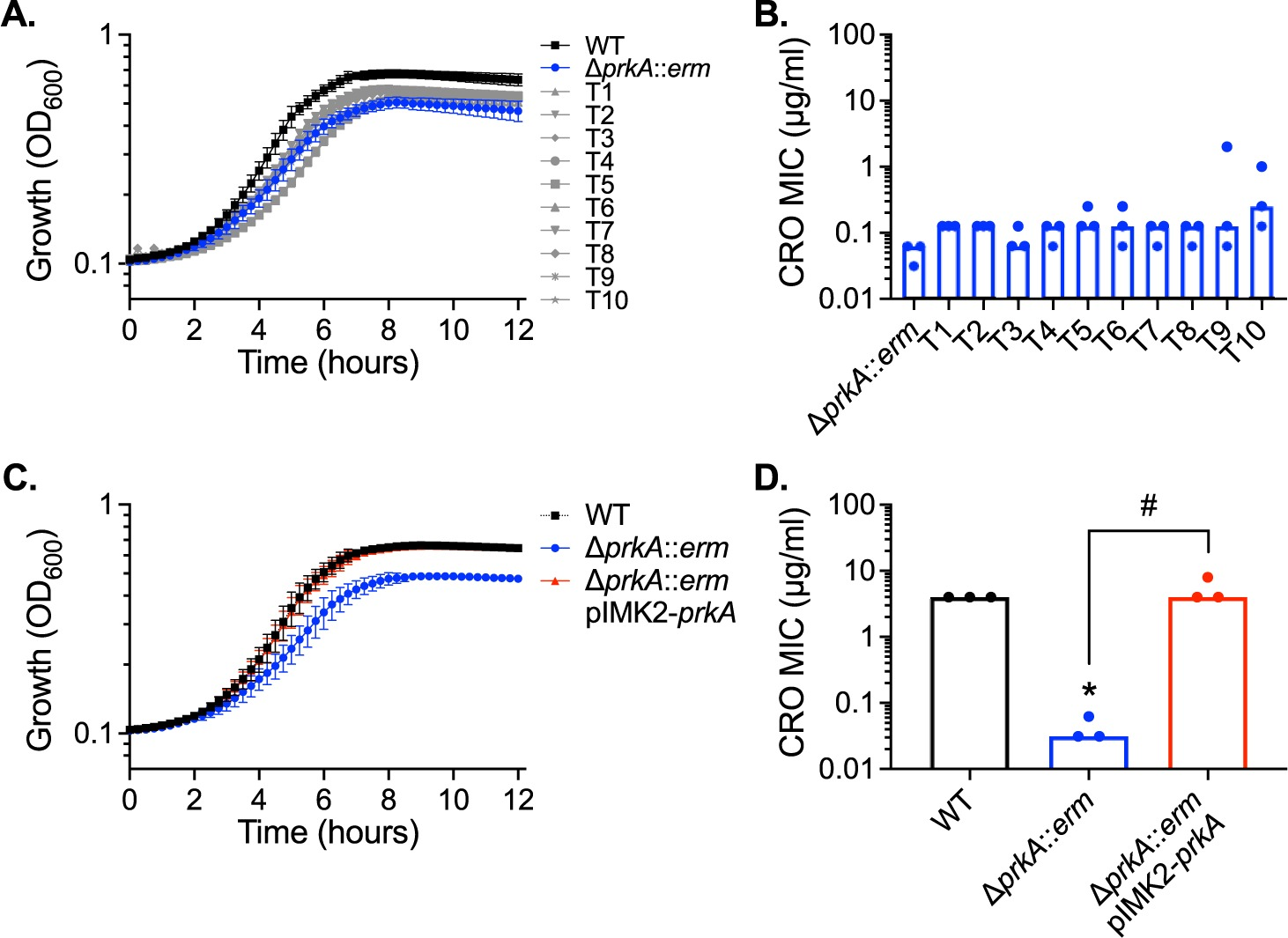

Supplement: S1 Fig — (A) Growth of WT, the ΔprkA::erm strain, and 10 ΔprkA::erm transductants (T1-T10) in BHI was monitored by OD600. Error bars indicate SD; n = 3. (B) Bars indicate median MICs of CRO for the indicated strains; n = 3. No statistical differences between strains were found between the transductants and ΔprkA::erm by one-way ANOVA with Tukey’s multiple comparisons test. (C) Growth of WT, the ΔprkA::erm mutant, and ΔprkA::erm carrying the pIMK2-prkA complementation construct in BHI was monitored by OD600. Error bars indicate SD; n = 3. (D) Bars indicate median MICs of CRO for the indicated strains; n = 3. *, P < 0.05 compared to wild type, and #, P < 0.05 for the indicated comparisons, by one-way ANOVA with Tukey’s multiple comparisons test. (TIF) [file ppat.1009881.s004.tif]

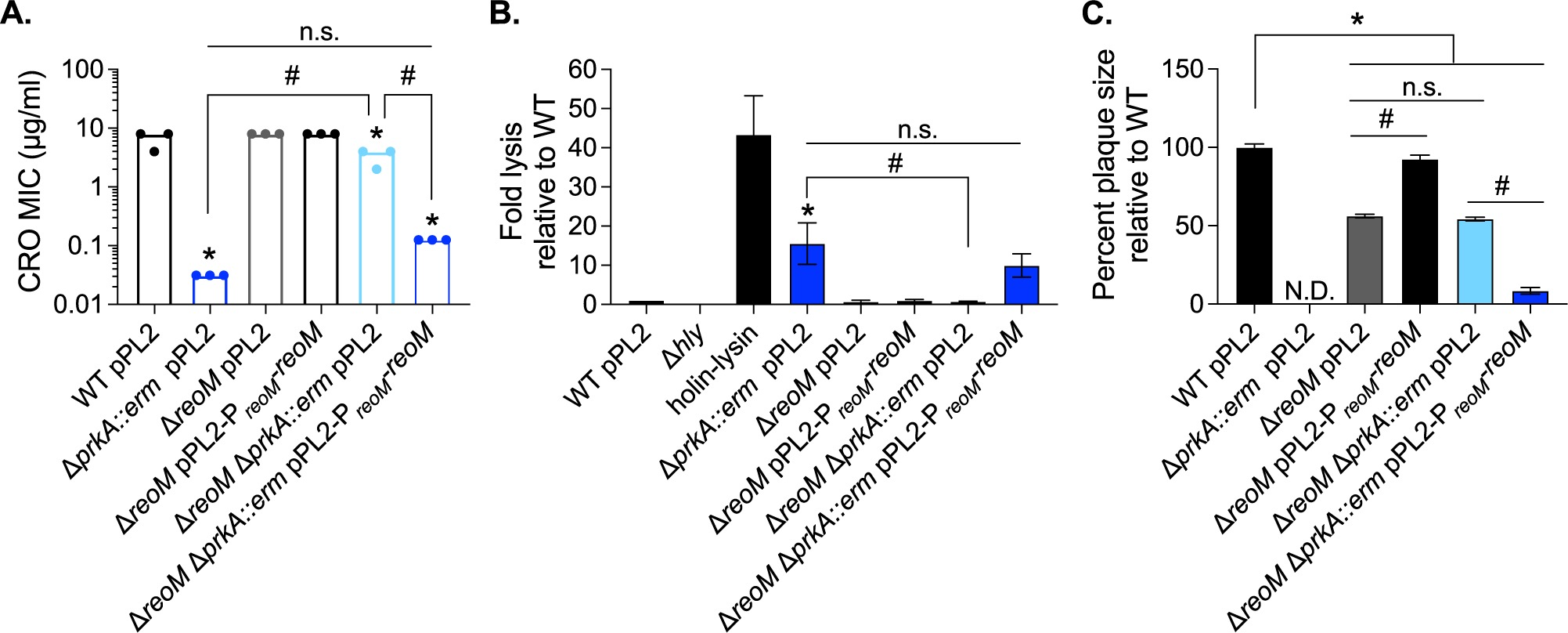

Supplement: S2 Fig — (A) Bars indicate median MICs of CRO for the indicated L. monocytogenes strains; n = 3. (B) Intracellular bacteriolysis in immortalized Ifnar-/- macrophages. Macrophages were infected with the indicated strains carrying the pBHE573 reporter vector at an MOI of 10, and luciferase activity was measured 6 hours post-infection. Error bars indicate SEM; n = 5. Plaque formation in immortalized murine fibroblasts (L2 cells). L2s were infected with the indicated strains at an MOI of ~0.5, plaques were stained on day 4 of infection, and sizes were normalized to those of wild type. Error bars indicate SEM; data are averaged from a minimum of 64 plaques from three biological replicates. N.D., not detected. (A-C) *, P < 0.05 compared to wild type, and #, P < 0.05 for the indicated comparisons, by one-way ANOVA with Tukey’s multiple comparisons test. (TIF) [file ppat.1009881.s005.tif]

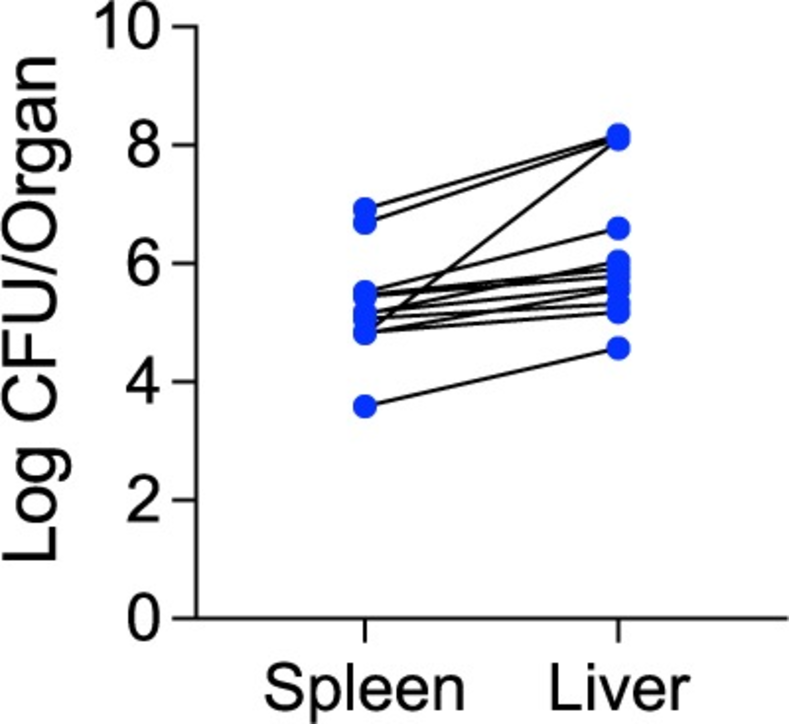

Supplement: S3 Fig — CFU were enumerated 72 hours post-infection with the EMS-mutagenized library of the ΔprkA::erm mutant. (TIF) [file ppat.1009881.s006.tif]
