# Supplementary material for: PASTA kinase-dependent control of peptidoglycan synthesis via ReoM is required for cell wall stress responses, cytosolic survival, and virulence in Listeria monocytogenes
Source: PLoS Pathog. 2021 Oct 8;17(10):e1009881. doi: 10.1371/journal.ppat.1009881 (PMC8528326; doi:10.1371/journal.ppat.1009881)
Supplement: Attachment — Submitted filename: Kelliher_PLOSPath_response_to_reviewers.docx [file ppat.1009881.s007.docx]

**Response to Reviewer Comments for Kelliher, JL *et al.***

We thank the reviewers for their thoughtful and constructive comments. Please find below our detailed response to specific concerns. We believe the manuscript is significantly strengthened as a result of the reviews.

***Part I – Summary***

*Please use this section to discuss strengths/weaknesses of study, novelty/significance, general execution and scholarship.*

*Reviewer #1: Using complementary methods, Kelliher et al. identify and validate ReoM as an important PrkA phosphosubstrate in vitro during peptidoglycan stress and likely in vivo. Their work raises fascinating questions about pathogen cell wall biogenesis and repair during different stages of infection, both at the cell and whole-animal levels. The experiments are rigorous and their claims are well-supported.*

We thank the reviewer for their positive comments.

*Reviewer #2: The manuscript by Kelliher and co-workers describes studies of the PrkA-ReoM signaling pathway in Listeria. They extend previous findings from both their group and that of Wamp et al to document that the PrkA-ReoM signaling pathway seems to be essential for infection and virulence by L. monocytogenes, and plays a role in antimicrobial resistance in S. aureus as well. They also report that the PrkA gene is not essential in L. monocytogenes, a finding that contradicts previous published reports. With a few exceptions described in detail below, the work is well done overall and the manuscript is clearly written.*

We thank the reviewer for their positive comments.

*Reviewer #3: The manuscript by Kelliher and co-workers titled “PASTA kinase-dependent control of peptidoglycan synthesis via ReoM is required for cell wall stress responses, cytosolic survival, and virulence in Listeria monocytogenes” is dealing with an important topic that involves regulation of peptidoglycan synthesis and its impact on virulence, in this case of the bacterial pathogen Listeria monocytogenes. The research was conducted by a group that carried out several studies on the impact of the serine/threonine associated kinase PrkA on L. monocytogenes virulence and cytosolic survival.*

*Unfortunately for this group, a paper was published in 2020 by Wamp et al., that similarly identifies ReoM as PrkA substrate. This paper also deciphered the function of these proteins in PG synthesis, demonstrating that PrkA and ReoM control the stability of MurA, which is an important enzyme in PG synthesis.*

*That said, the study performed here is beautiful, combining phosphoproteomic and genetic screens to search for PrkA substrates (under cell wall stress). Moreover, this study, in contrast to the one by Wamp, shows the effect of PrkA and ReoM on L. monocytogenes virulence. The paper is very well written and the results support the main conclusions. It provides new information regarding the regulation of PG synthesis during Lm infection of mammalian cells, and hence to my opinion justifies a publication in PLoS Pathogens.*

We thank the reviewer for their positive comments.

***Part II – Major Issues: Key Experiments Required for Acceptance***

*Please use this section to detail the key new experiments or modifications of existing experiments that should be absolutely required to validate study conclusions.*

*Generally, there should be no more than 3 such required experiments or major modifications for a "Major Revision" recommendation. If more than 3 experiments are necessary to validate the study conclusions, then you are encouraged to recommend "Reject".*

*Reviewer #1: No major issues*

*Reviewer #2: Figure 3, and lines 323, 342, 350-1, 352-3, 354-5, 491-3 and elsewhere: The authors use an innovative click-chemistry technology for labeling of PG of different strains in the presence/absence of antimicrobial treatment. Although this labeling technique is powerful, my main concern is that the authors equate the extent of such labeling to the amount of PG synthesis, and this interpretation does not seem justified given the available data. The labeling strategy can provide insights into “where” in the cell the PG is, or “when” it is synthesized (depending on the experimental setup), but extending it to “how much” PG is synthesized is a bit tricky. This is largely due to the fact that PG is dynamic, and the amount of PG at any given time (i.e. the amount of labeling observed) depends on both how much is made as well as the turnover (i.e. loss) as well as the efficiency of label incorporation. For example, if the label is incorporated more efficiently into nascent PG under a given set of conditions (e.g. a mutant strain, or drug treatment), then the labeling signal will increase even if the amount of PG has not. There are numerous reasons that the efficiency of label incorporation could increase (e.g. enhanced turnover of existing PG through hydrolytic enzymes, enhanced uptake of metabolic label into cells, etc), and these have not been experimentally addressed in the manuscript. In short, the authors stated conclusions that prkA / reoM control the amount of PG synthesis is one plausible possibility, but it is not rigorously supported by the available data.*

We appreciate the reviewer’s thoughtful concerns about this experiment. We agree that the dynamic nature of PG synthesis and turnover makes it difficult to ascribe these changes to differences in synthesis exclusively. We have changed the language in the corresponding results section (lines 350-371) and added a sentence to the discussion (lines 504-507) to reflect this.

*Figure 3: Related to the above point, the growth kinetics / growth stage of the strains for the PG labeling study is not clear from the description given, and could impact the outcome. Do all the strains analyzed grow at the same rate? Were they in the same stage of growth at the time of sampling? Although it is a bit hard to tell, based on the growth curve shown in Supp Fig 1 and the description in the methods (lines 765-7), one might imagine the strains (untreated wild-type, at least) would nearly be entering stationary phase at the time of sampling/harvest, which complicates things because it might be expected that PG synthesis would be slowing during that time. If the drug-treated cells or mutant strain is growing more slowly and not yet entering stationary phase at the time of sampling, PG synthesis could be inherently higher simply for that reason.*

The growth curve shown in Suppl. Fig. 1 was performed in a 96-well plate in a microplate reader while the PG labeling experiments were done in culture tubes on a shaker platform, so the OD_600_ values cannot be directly compared between the two experiments as the tubes promote more aerobic growth than the 96-well plate. As stated in the methods, bacteria were diluted to OD 0.3 from exponentially growing cultures and incubated with shaking for 1 hour in the absence or presence of ceftriaxone. All samples increased in OD during that time (from 0.3 to 0.5-0.9, in early to mid-log phase), indicating all samples grew, but had not reached stationary phase. In these growth conditions the cultures will reach a maximum OD600 of ~3.5 making the point of sampling well before the entry to stationary phase. We have added a statement to the methods clarifying that the cultures were all sampled in early to mid-exponential phase (lines 785-786). The addition of half an MIC of ceftriaxone did not have a marked effect on growth, i.e., strains grew similarly +/- drug.

*line 393: This statement is a bit strongly worded given the data presented. Strictly speaking, the authors analyzed strains lacking reoM or prkA entirely, and did not specifically demonstrate that ReoM is phosphorylated (by PrkA or otherwise) during infection. While it is clear that loss of reoM has phenotypic consequences during infection, based on this data it does not seem justified to specifically attribute those consequences to PrkA-dependent phosphorylation of ReoM during infection. A similar argument applies to the S. aureus data and conclusion (line 433).*

We appreciate the reviewer’s comment and agree that we have not demonstrated phosphorylation of ReoM during infection. Wamp *et al.* have shown through a combination of targeted genetics and mass spec approaches that PrkA phosphorylates ReoM in *Lmo* during cell wall stress. Wamp *et al.* further showed that mutation of the ReoM phosphosite T7 to a non-phosphorylatable alanine was sufficient to sensitize *Lmo* to ceftriaxone, suggesting that PrkA phosphorylation of ReoM is the critical interaction between these two proteins. Although we agree they are not conclusive, on the whole, our genetic data in *ex vivo* and *in vivo* models support this model as well. We have updated language in the discussion section (lines 567-571) and elsewhere in the manuscript (lines 404, 440, 444, 458, 1215) to reflect these ideas.

*Reviewer #3: I don't have such experiments.*

***Part III – Minor Issues: Editorial and Data Presentation Modifications***

*Please use this section for editorial suggestions as well as relatively minor modifications of existing data that would enhance clarity.*

*Reviewer #1:*

*• Author Summary has technical language that should be simplified and/or explained more, e.g., phosphoproteomics*

We have updated the summary to use simplified language (lines 44-58).

*• P. 11 lines 237-238: I believe the samples were harvested only from drug-treated wild-type and prkA mutant. In the absence of untreated controls, it is not quite accurate to say that PrkA mediates a global response to cell wall stress since we do not know what wt vs. mutant looks like in the absence of that stress.*

We have changed the wording to clarify (line 239).

*• P. 12 line 266: I know there is a reference, but it would be helpful to the reader to include a very brief summary of how the reporter system works.*

We have added an explanation of the reporter system (lines 267-270).

*• P. 13/14 lines 288-290: I think it’s a bit more nuanced than the concluding sentence implies. Suppressor phenotypes in panels A and B correlate well but less true for panels D and E. This is explained well in the preceding paragraph, although I disagree that the parent prkA mutant is “cleared” in Fig. 2D as described in line 276.*

We appreciate this concern and have updated the language in the manuscript to reflect this (lines 281-282, 285-286, 294, 303-306, references 60 and 61).

*• Fig. 3B labels hard to read.*

We have updated Fig. 3B to improve clarity.

*• P. 26 lines 569-570: likely to be true but phosphoproteomics performed in vitro*

Please see above our response to Reviewer #2.

*• A model cartoon would augment the discussion. I see that Wamp et al. have one, but it would be helpful to visualize the signaling pathway within a broader, host-pathogen context, especially since this is a key aspect of the current work.*

We appreciate the suggestion and have added a model cartoon as Figure 7 (lines 467, 1226-1244).

*Reviewer #2: lines 197-211: The conclusion that prkA is not essential is based on reasonably compelling analyses (whole genome sequencing and phage transduction studies). But, whole genome sequencing can miss genomic variants due to gaps or low coverage in some regions, and phage transduction could co-transduce a secondary mutation that is physically close to the prkA::erm allele in the genome. Given the contradiction of the current results with those published in the literature previously (i.e. that prkA is essential), why not just do a complementation assay by expressing prkA in trans in the prkA::erm mutant to demonstrate a rescue of the growth defect and put the final nail in the coffin on this? It appears the authors possess a plasmid with inducible prkA that would allow them to do this.*

We agree that testing for complementation is important and have added this data as Supplemental Figures 1C-D (lines 195-197, 616-619, 1295-1303, Tables 6-8, reference 74). Expression of *prkA* from a constitutive promoter *in trans* complements both the growth defect in BHI and the sensitivity to CRO of a Δ*prkA*::*erm* mutant. With respect to genome coverage, our sequencing depth averaged 65 reads per base across all sequenced genomes, and there was no obvious decrease in coverage within 10 kb of the *prkA* locus, suggesting that we should have been able to identify mutations close to the *prkA*::*erm* allele.

*Figure 6: Would have been nice to see some complementation data for the S. aureus mutants described here.*

We appreciate the reviewer’s comment. We think that showing that mutations in three independent genes in the ReoM pathway, *reoM*, *reoY*, and *clpP*, are able to reverse the sensitivity of *stk1* mutants to oxacillin supports our conclusion that this pathway functions similarly in *S. aureus* as it does in *Lmo*. In addition, the strains were constructed in two different ways: for the *stk1*::*erm* Δ*reoM* mutant, the Δ*reoM* mutation was made first and the *stk1* allele subsequently transduced in. For the Δ*stk1 reoY*::*erm* and Δ*stk1*::*clpP* mutants, *reoY* and *clpP* lesions were transduced into a clean Δ*stk1* background. Altogether, independent methods of strain construction and consistency across multiple mutants in the pathway suggest there are not compensatory mutations in these strains.

*Tables 4 and 5: It is unclear how nucleotide variants identified in intergenic regions can be assigned to particular genes without experimental analysis (e.g. PCS1 murZ [Table 4], PEVS3 guaA [Table 5], others). For example, it isn’t hard to imagine that such intergenic variants could affect some regulatory element (e.g. small RNA) that is not related to the adjacent gene.*

We understand the reviewer’s concern. In all these cases, the SNPs were upstream of the gene to which we assigned it, and the next gene upstream is encoded in the same direction. We have added details about the location of the SNP relative to the start codon of the assigned gene (Tables 4 and 5). We do agree that there is a possibility that these SNPs may affect a regulatory element that unrelated to the adjacent gene and have added that idea to the text (lines 320-322). However, in the case of *reoM* and *murZ*, because Wamp *et al.* have shown that deletion of *reoM* or *murZ* increases MurA levels and have previously published that a SNP in the RBS of *reoM* restores MurA levels, we think it is appropriate to correlate intergenic SNPs with decreased expression of these genes and rescue of *prkA* phenotypes; we have clarified this in the text (lines 308-310).

*Reviewer #3: Minor comments:*

*In order to better deduce the role of PrkA in phosphorylation of target proteins, the authors performed a comprehensive analysis of multiple phosphosites of L. monocytogenes proteins. I expect this data to be interesting to researchers dealing with Listeria, and hence I would like to recommend the authors to slightly edit the presentation of both Supplementary Tables (1 and 2). Currently the data within these tables are associated with the use of Reference Proteins whose accession numbers have a universal prefix WP_. From my experience, it is not that intuitive to ‘translate’ any WP_ accession number to a real gene tag of the 10403S genome as well as to that of strain EGDe. It would be useful if the authors can add an additional column that provides real gene tags of strains 10403S and EGDe.*

We appreciate this suggestion and have added 10403S and EGD-e gene tags to all Supplementary Tables.

*I wonder if the authors can comment on the possible roles of the other two proteins encoded by the operon of reoM. lmo1502 (LMRG_01468) and lmo1501 (LMRG_01469) located immediately downstream to reoM (lmo1503, LMRG_01467). Have the authors analyzed any feedback regulatory changes of these genes in the delta reoM mutant? According to Wurtzel et al (2012) this operon has a high expression level and it would be interesting to learn how it is regulated? lmo1502 encodes a protein resembling RNase H, while lmo1501 encodes a conserved acidic protein (pI 3.59) of unknown function (DUF1292; pfam06949). Are they important for ReoM expression/activity?*

While we share the reviewer’s enthusiasm for understanding more about ReoM and agree examining other members of its operon would be an excellent approach, we believe such analysis is outside of the scope of this manuscript and have not yet followed up on *lmo1502* or *lmo1501*. Since we generated a clean deletion of *reoM* and we were able to complement Δ*reoM* phenotypes with expression of only *reoM*, we presume that expression of other members of this operon is minimally disturbed in the Δ*reoM* background.

*I would suggest another presentation of the PSC results; first sequencing the mutants, then basic characterization of all mutants, and then focus on three mutants of your choice.*

We appreciate this idea. We randomly selected the 3 PCS mutants to characterize phenotypically and sequenced those genomes first. Once we sequenced the remaining 9 mutants, given that all had mutations in the ReoM pathway, we did not further characterize the latter 9 phenotypically. If we had sequenced all PCS mutants first, we feel it would be difficult to justify having picked 3 PCS mutants with multiple mutations as opposed to the two (PCS7 and PCS20) with only one mutation relative to the parent Δ*prkA*. Therefore, we don’t think we have quite the right data to support this suggested rearrangement.

*Figure 3B- unreadable and should be presented as a table.*

As noted above, we have updated Fig. 3B to improve clarity.
